# The impact of healthy motion seating on lower‐limb blood flow and blood pressure response to simulated long‐haul air travel

**DOI:** 10.1113/EP092920

**Published:** 2025-11-04

**Authors:** Jane Lewis, Barry J. Mcdonnell, Mark Butlin, Edward Johnston, Amira Tairi, Thomas Griffiths, Gisele Bentley, Peter Sykes, Keeron Stone

**Affiliations:** ^1^ Centre for Cardiovascular Research, Innovation and Development (CURIAD), Cardiff School of Sport and Health Sciences Cardiff Metropolitan University Cardiff UK; ^2^ National Cardiovascular Research Network Cardiff Wales UK; ^3^ Faculty of Medicine, Health and Human Sciences Macquarie University Sydney Australia; ^4^ College of Biomedical and Life Sciences Cardiff University Cardiff UK; ^5^ Faculty of Medicine Université Laval Quebec Canada

**Keywords:** aerospace, mood, pain, prolonged sitting, pulse wave analysis, sedentary behaviour

## Abstract

Prolonged sitting inherent to long‐haul air travel can acutely decrease lower‐limb blood flow and increase brachial blood pressure. Healthy motion seating (HMS), which passively alters sitting interface pressure and posture, is a promising technology which may attenuate the deleterious effects of long‐haul air travel. The aim of this study was to determine the impact of an innovative airplane passenger seat motion feature, aerospace HMS, on lower‐limb blood flow and blood pressure in response to 6.5 h of simulated long‐haul air travel. In a randomised cross‐over design, 19 healthy adults completed a 6.5 h long‐haul flight simulation in aerospace seating equipped with (HMS) and without (CON) healthy motion technology. Superficial femoral artery blood flow, brachial blood pressure, and perceptions of mood disturbance, pain and discomfort were measured before and after flight simulation. In linear mixed models there was a significant interaction (condition × time) effect for superficial femoral artery blood flow, with a decrease in lower‐limb blood flow in CON (−22.4 mL/min; 95% CI: −2.41, −42.39; *P* = 0.032) but not HMS (3.7 mL/min; 95% CI: −16.3, 23.67; *P* = 0.720) across the 6.5 h flight simulation. There were no interaction, group nor time effects for blood pressure. Mood, pain and discomfort all worsened across the 6.5 h flight simulation (time, all *P *< 0.05), but there were no interaction nor group effects. The passive alterations in sitting interface pressure, posture and movement created by aerospace HMS can prevent prolonged sitting‐induced reductions in local lower‐limb blood flow typical of long‐haul air travel.

## Abstract (highlights)

1. **What is the central question of this study?**
  What is the impact of an innovative airplane passenger seat feature, aerospace healthy motion seating, on lower‐limb blood flow and brachial blood pressure in response to 6.5 h of simulated long‐haul air travel?
2. **What is the main finding and its importance?**
  The passive periodic alterations in sitting interface pressure, posture and movement created by aerospace healthy motion seating were protective against reductions in local lower‐limb blood flow during simulated long‐haul air travel. Use of healthy motion seating has the potential to enhance the well‐being and experience of passengers during long‐haul air travel and may be particularly relevant to those with compromised cardiovascular health.

## 1 INTRODUCTION

Air travel, particularly long‐haul flights, is inherently associated with travellers having to spend an extended period participating in sedentary behaviours, defined as a seated, reclined or lying posture and low energy expenditure (Tremblay et al., 2017). Sedentary behaviour, typically prolonged sitting, has been shown to impair cardiovascular health, notably vascular function in the lower limbs (Paterson et al., 2020) and brachial blood pressure (Paterson et al., 2022). This vascular dysfunction is attributed to reduced blood flow and shear stress (Restaino et al., 2016; Thosar et al., 2012), whilst reduced metabolic demand and heightened sympathetic nervous system activity further elevates blood pressure (Dempsey et al., 2018; Dunstan et al., 2021). Prolonged sitting can also worsen mood (Carter et al., 2021) and provoke musculoskeletal discomfort and pain (Benzo et al., 2018). Regularly interrupting prolonged sitting with light physical activity can help preserve lower‐limb blood flow and brachial blood pressure (Paterson et al., 2022, 2020), and attenuate symptoms of discomfort, pain (Gallagher et al., 2019) and mood disturbance (Bergouignan et al., 2016). Notwithstanding, the limited space on aircraft makes physical activity interruptions logistically impractical and inconvenient, whilst many passengers prefer to remain seated. Attention has focussed on improving aerospace seating ergonomics (Porta et al., 2019; Sadai & Gilad, 2015; Smulders et al., 2016), with one promising concept being the use of seating equipped with motion technology which frequently and automatically changes a seats position to generate changes in a passenger's posture, termed healthy motion seating (HMS).

Research evaluating HMS has predominantly been undertaken by the automobile industry, where the focus has been to optimise pressure load distribution and instigate postural movements to improve comfort (Aota et al., 2007; Reinecke et al., 1994). Compared to standard static automobile seating, the periodic movement and subsequent shift in sitting posture (extension and flexion of the hip and knees, raising and lowering the torso and legs) and pressures (seat pan to/from seat back) generated by automobile HMS reduced muscle fatigue in the lumbar muscles (Tepe & Zhang, 2011) and attenuated perceptions of musculoskeletal pain and mood disturbance (Dugan et al., 2009) induced by 3 h of prolonged sitting. But there is little understanding of the haemodynamic impact of HMS. It has been reported that automobile HMS can also attenuate sitting‐induced reductions in blood flow velocity in the lower limbs (Dugan et al., 2009), but the use of a short sitting duration (∼34 min) and lack of vessel diameter measurement (needed to characterise flow) means that this finding offers only partial insight into whether HMS can positively impact lower‐limb blood flow dynamics during longer bouts of sitting. Changes in sitting interface pressure (Zemp et al., 2019) and posture (Antle et al., 2018), as well as passive movement (Trinity et al., 2011), are all known to impact both lower‐limb blood flow and brachial blood pressure. Whether periodic shifting of these states, as that which occurs with HMS, can prevent the reductions in lower‐limb blood flow and increases in blood pressure expected in response to prolonged sitting remains unclear. Further, if HMS is to be of utility in the aerospace industry, there is a need to identify whether it is effective in combatting the impact of prolonged sitting within the unique aerospace seating environment and extended time periods of long‐haul air travel. Therefore, the aim of this study was to determine the impact of an innovative airplane passenger seat feature, aerospace HMS, on lower‐limb blood flow and brachial blood pressure in response to 6.5 h of simulated long‐haul air travel.

## 2 METHODS

### 2.1 Ethical approval

Study procedures were approved by the ethical committee of Cardiff School of Sport and Health Sciences under the Cardiff Metropolitan University Ethics Framework (Reference: STA‐7116). This study is reported according to the Consolidated Standards of Reporting Trials (CONSORT) guidelines (Schulz et al., 2010). All study participants provided written informed consent before participation. The study conformed to the standard set by the *Declaration of Helsinki*, except for registration in a database.

### 2.2 Study design

The study comprised a randomized cross‐over design, with two counterbalanced arms: a simulated flight in a standard aerospace static seat (CON), and a simulated flight in an aerospace seat equipped with HMS technology. Each simulated flight lasted 6.5 h and was chosen because this time represents the minimum duration for what is regarded as a long‐haul flight. A minimum of 2 days separated each visit to minimize carry‐over effects, with a maximum of 7 days to minimize within‐subject variation. The sequence of interventions was randomly allocated using Research Randomiser (https://www.randomizer.org/; Urbaniak & Plouse, 2013), which is based on the ‘Math.random’ method within the JavaScript programming language.

### 2.3 Participants

A healthy population sample was recruited to mitigate the risk of age or disease‐related influences on the primary outcomes. Participants were included if they were free of overt cardiovascular disease and were aged between 18 and 80 years. Participants were excluded if they reported any known cardio‐metabolic disorders, were taking medications known to affect cardiovascular function, reported cigarette smoking or vaping, or were pregnant.

### 2.4 Experimental protocol

Prior to each experimental visit, participants were asked to refrain from drinking alcohol or performing strenuous physical activity for at least 24 h, and refrain from caffeine for at least 12 h. Participants were asked to consume a light breakfast at least 2 h prior to arrival, which was replicated between visits. Visits started between 08.00 and 09.00 h and each participant arrived at the same time for both visits. Upon arrival participants were asked to fully void their bladder if possible.

**FIGURE 1:**
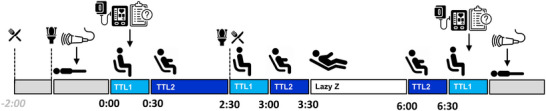
Schematic representation of the experimental protocol for the 6.5 h simulated air travel.

Figure 1 provides an overview of the sitting protocol employed for each experimental arm. With the seat fully reclined, participants were asked to lie in a supine position for 15 min during which they were instrumented. Following this, baseline measures of superficial femoral artery blood flow were taken. Participants were then moved into a seated posture and, following 5 min rest, seated measures of brachial blood pressure (BP) and pulse wave analysis (PWA), and calf circumference were taken. Immediately following this, participants completed the short‐form McGill pain, the profile of mood states (POMS), and the Mansfield 2‐part comfort questionnaires. Participants then commenced a 6.5 h prolonged sitting protocol, during which time the seat automatically moved between three different postural positions: (1) taxi‐take‐off and landing 1 (TTL1, i.e. seated); (2), taxi‐take‐off and landing 2 (TTL2, i.e. reclined with limited leg rest); and (3) lounge (i.e. semi‐recumbent with extended leg rest). Seated measures of BP, PWA and questionnaires were repeated at 06.30 h. Participants were passively transitioned to a supine posture using the seat's integrated actuation system, and superficial femoral artery blood flow was measured after 15 min of rest.

To replace predicted water loss, 1.5 L of water was provided for participants to consume ad libitum during the sitting protocol. At 02.30 h, participants were permitted to take a 5‐min comfort break, following which they were provided with a standardised lunch that is typically served to business and first‐class passengers for a long‐haul flight, and met recent dietary guidelines (Snetselaar et al., 2021). This meal was repeated for the second experimental visit. The researchers did not prescribe activities during the sitting protocol; participants were allowed to read, watch television or use a laptop akin to activities typical of a long‐haul flight. Apart from the specified mid protocol (∼02.30 h) comfort break, there were no instances of participants getting up for any reasons throughout the sitting protocol. The experimental procedures were conducted in a quiet and environmentally controlled room (20.5 ± 0.8°C), with low‐lighting conditions akin to long‐haul flights.

### 2.5 Healthy motion seating

The experimental protocol utilised four Thompson Aero Seating ‘Vantage’ seats commonly used in business‐class air travel. Two seats were used as controls having no modifications and two seats were modified to incorporate the HMS technology. The modifications comprised replacing the existing actuation system (the electronics control unit and seat actuation motors) with an Astronics (New York, NY, USA) PGA Gen V11 actuation system, which was re‐programmed with an algorithm that permits slow and smooth seat position changes. During the 6.5 h simulated flight sequence in the experimental arm (HMS), participants experienced HMS for a total of 5.5 h in two different seat positions: (1) taxi, take‐off and landing 2 (TTL2), and (2) lounge (Lazy Z) (Figure 1). In the TTL2 home position, the seat back is slightly reclined, the seat pan is slightly forward, and the leg rest is not deployed. In the Lazy Z home position, the seat back is significantly reclined, the seat pan is forward and slightly raised, and the leg rest is deployed to support the lower legs. From these home positions the HMS algorithm adjusts the seat back and leg rest positions. The HMS algorithm is fully described in Supporting information, Tables S1–S3.

### 2.6 Experimental measures

#### 2.6.1 Superficial femoral artery blood flow

An ultrasound device (T3300, Terrason, Burlington, MA, USA) fitted with a linear array probe (15–4 mHz) was used to simultaneously measure blood flow velocity and diameter of the superficial femoral artery. Blood flow was determined in the supine position to allow better access to the superficial femoral artery's acoustic window. Three 10 s videos of the superficial femoral artery were recorded on the right side of the body and were captured at 20 Hz using screen recording software (Camtasia, TechSmith, Okemos, MI, USA) inherent to the ultrasound device. The captured videos were analysed offline using custom‐designed automated edge‐detection wall‐tracking and velocity waveform tracking software (FMD/BloodFlow Software v1.1, Reed Electronics, Perth, WA, Australia), the validity and reproducibility of which has been demonstrated (Woodman et al., 2001). Custom written Microsoft Excel Visual Basic code was used to fit peaks and troughs to the diameter waveforms to calculate diastolic, systolic and mean diameters (Stoner & McCully, 2012; Stoner et al., 2020). Blood flow was calculated from continuous diameter and mean blood velocity recordings using the following equation: 3.14 × (diameter/2)^2^ × mean blood velocity × 60. Shear rate (s^−1^) was calculated as 4 × mean blood flow velocity/blood vessel diameter. The mean of the closest two measures from the three 10 s recorded sequences was used in analyses.

#### 2.6.2 Brachial blood pressure and pulse wave analysis

The Mobil‐O‐Graph system (IEM Germany, Stolberg, Germany) was used to non‐invasively measure brachial blood pressure and undertake pulse wave analysis to determine central haemodynamic indices. The Mobil‐O‐Graph system has been validated for ambulatory blood pressure measures according to the British Hypertension Society, with comparison to criterion devices and invasive arterial blood pressure monitoring (Benas et al., 2019; Franssen & Imholz, 2010; Weber et al., 2011). In brief, conventional oscillometric blood pressure measurement was first completed on the upper arm to determine brachial systolic (SBP) and diastolic (DBP) BP. Subsequently, a 10 s sub‐diastolic recording of brachial pulse waves was completed, from which a corresponding aortic waveform was generated using the validated (ArcSolver) transfer function. From this sub‐diastolic recording, central systolic blood pressure (cSBP), central augmentation pressure (cAP), augmentation index (AIx), augmentation index normalized to a heart rate of 75 bpm (AIx@75), and aortic pulse wave velocity (aPWV) were derived. Stroke volume (SV), cardiac output (CO) and systemic vascular resistance (SVR) were also determined (Weber et al., 2011). Before and after the sitting 6.5 h protocol, in supine and seated positions, all BP and PWA assessments were conducted in triplicate and separated by 1 min, with the mean of the closest two used for all analyses.

#### 2.6.3 Mood, pain and comfort

The Profile of Mood States (POMS) is a 65 item multi‐dimensional Likert self‐reporting scale typically used to assess transient and fluctuating mood states. This POMS scale consists of 65 adjectives, which the respondent ranks on a scale of ‘Not at All’, ‘A little’, ‘Moderate’, ‘Quite a Lot’ and ‘Extremely’. Six subscales are derived from these adjectives and assess tension–anxiety, depression, anger–hostility, fatigue, confusion–bewilderment, and vigour–activity. From this Total Mood Disturbance, a global index of distress, can be calculated from the respective subscale scores. The POMS is a valid self‐report assessment of mood that is adaptable for capturing transient and fluctuating feelings in adults aged over 18 years (Terry et al., 2003). The Short Form McGill Pain Questionnaire 2 (SF‐MPQ‐2) is a 22‐item self‐reporting questionnaire that provides a single measure to quantify both neuropathic and non‐neuropathic pain by evaluating the importance of the sensory, affective and evaluative aspects of pain and pain intensity on a scale of 0 (None) and 10 (Worst Possible Pain) (Dworkin et al., 2015). The 22 descriptors establish the following four subscales: continuous pain, intermittent pain, neuropathic pain and affective descriptors. The pain visual analogue scale (VAS), part of the original SF‐MPQ, can be used as a subjective unidimensional measure of overall pain intensity (Hawker et al., 2011). The SF‐MPQ‐2 has been validated for use in adults with acute musculoskeletal pain (Dworkin et al., 2015). Part 1 of the Mansfield 2‐part Comfort Questionnaire (MCQ) is a six‐point Discomfort Scale, which provides a measure assessing the effects of an intervention on discrete body regions, namely upper back, lower back, sitting bones, buttocks area and edge of seat contact. The MCG is a preferred comfort questionnaire for comparing two products, as recommended by the International Comfort Congress (Anjani et al., 2021; Sammonds et al., 2017).

#### 2.6.4 Sample size estimation

Prior literature seeking to compare the impact of prolonged uninterrupted sitting (>3 h) with sitting interrupted with local (fidgeting) or light (standing, walking) physical activity (the closest equivalent to the HMS technology) on lower‐limb blood flow and blood pressure has utilised sample sizes ranging from 8 to 20 (Carter et al., 2019; Fryer et al., 2023; Paterson et al., 2022, 2020), with a median of 12. Based on this prior work, we calculated that 18 subjects would be required to detect a moderate effect change in lower‐limb blood flow, with an assumed correlation between measurements of 0.7, an alpha error probability of 0.1 and 80% power. A recruitment target of 22 participants was chosen to account for likely drop‐out based upon our prior work and potential missing data.

### 2.7 Statistical analysis

Statistical analyses were performed using Jamovi 2.2.5, an open‐source statistical program based on R coding. Only participants with complete data on primary outcomes were included in the analyses. The effects of time (pre vs. post) and condition (CON vs. HMS) were analysed using linear mixed models, with the participant (intercept) specified as a random effect and time (slope) and condition specified as fixed effects. A statistical threshold of *P* = 0.10 was used to evaluate time × condition interaction effects to account for reduced statistical power (Matuschek et al., 2017), whereas a threshold of *P* = 0.05 was used for evaluation of main effects of time or condition. To limit within‐subject variance, the models were adjusted for baseline values. Effect sizes (ES) were calculated as Cohen's *d* by dividing β by the standard deviation, where <0.2, 0.2–0.5, 0.5–0.8 and >0.8 were defined as trivial, small, moderate and large, respectively (Hopkins et al., 2009). Raw data are presented as means (SD), and mixed‐model data are presented as means and 95% confidence intervals (95% CI).

## 3 RESULTS

Twenty‐four healthy adults were recruited to participate in the study. Five participants (3 males, 2 females) completed one experimental visit but withdrew from the study due to scheduling issues (four) or illness (one). Demographic characteristics of those participants who withdrew from the study did not differ from those included in our analysis (age: 35 ± 7 years; body mass index: 26.9 ± 7.2 kg/m^2^; SBP: 114 ± 4 mmHg; DBP: 69 ± 5 mmHg). Complete data were collected on 19 participants (11 male, 8 female) with a mean age of 36 ± 15 years, mean body mass index of 25.2 ± 5.9 kg/m^2^, and a resting HR, SBP and DBP of 68 ± 8 bpm, 119 ± 19 mmHg and 74 ± 8 mmHg, respectively.

**FIGURE 2:**
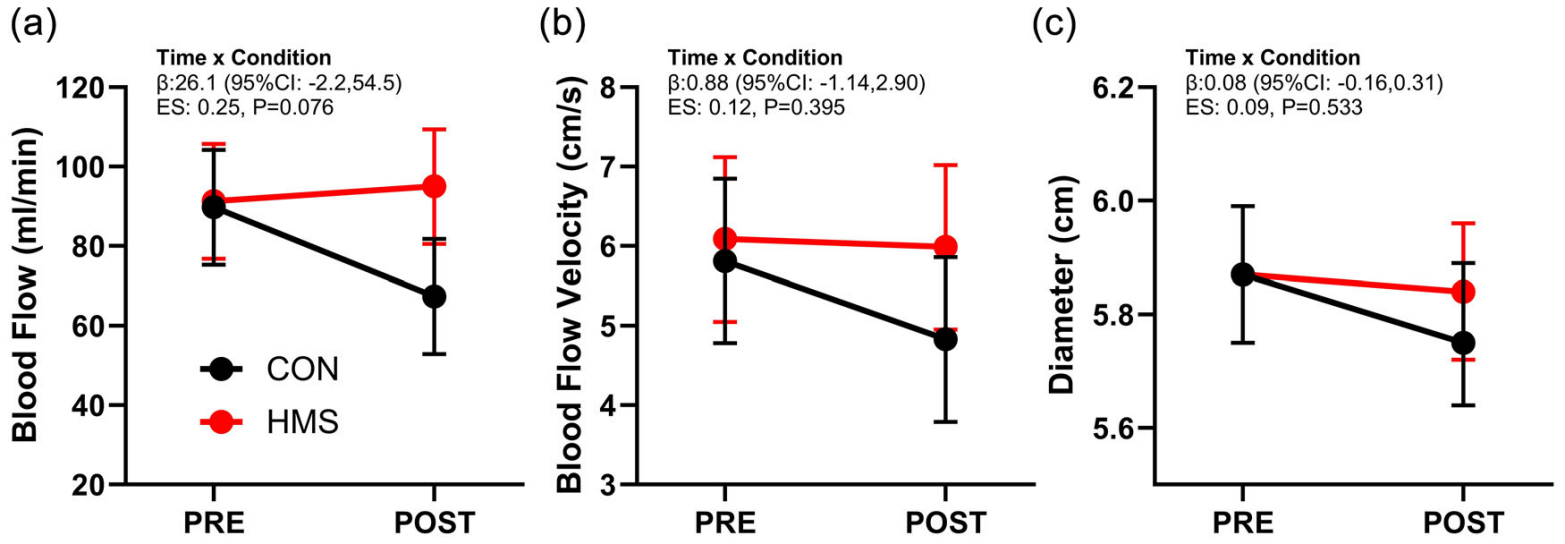
Supine superficial femoral artery mean blood flow (a), blood flow velocity (b), and diameter (c) responses to the 6.5 h flight simulation in control seating (CON) and Healthy Motion Seating (HMS). Data presented are means and 95% CI and are derived from baseline adjusted mixed models.

Raw superficial femoral artery blood flow data are presented in Table 1. Baseline adjusted mixed model superficial femoral artery blood flow data are presented in Figure 2. There was a significant interaction effect (time × condition, *P* = 0.076) for mean superficial femoral artery blood flow only, with a small effect decrease for CON (−22.4 mL/min; 95% CI = −2.41, −42.39; ES 0.31, *P* = 0.032) and no change for HMS (3.7 mL/min; 95% CI = −16.3 to 23.67; ES 0.05, *P* = 0.702) across the 6.5 h flight simulation (Figure 2a). There was a small time effect for minimum blood flow velocity indicating an increase in retrograde blood flow velocity across the flight simulation (−2.29 cm/s; 95% CI = −4.10, −0.47; *P* = 0.017, ES −0.35). Raw superficial blood pressure and pulse wave analysis data are presented in Table 2. There were no significant interaction, group nor time effects for any of the blood pressure or pulse wave analysis variables in response to the 6.5 h flight simulation.

**TABLE 1:** Supine superficial femoral artery blood flow responses to the 6.5 h flight simulation in control‐static seating (CON) and Healthy Motion Seating (HMS).

		*D*_dia_ (mm)	*D*_sys_ (mm)	*D*_mean_ (mm)	*V*_min_ (cm/s)	*V*_max_ (cm/s)	*V*_mean_ (cm/s)	BF_mean_ (ml/min)	SR (1/S)
Mean									
CON	PRE	5.8	6.1	5.9	−33.7	66.6	5.5	87.1	38.5
	POST	5.7	6.0	5.8	−35.3	67.5	4.5	64.7	33.3
EXP	PRE	5.7	6.0	5.8	−33.8	69.5	6.4	94.0	45.9
	POST	5.6	6.0	5.8	−36.7	73.2	6.3	97.7	44.4
Standard deviation									
CON	PRE	1.1	1.1	1.1	7.4	8.1	2.4	42.2	18.6
	POST	1.1	1.1	1.1	9.8	8.8	2.7	31.3	22.8
EXP	PRE	1.0	1.1	1.0	8.7	7.8	2.8	37.5	23.1
	POST	1.0	1.1	1.1	7.5	12.4	3.3	58.8	24.9
Condition effect									
β		0.02	0.05	0.04	−0.68	1.90	0.72	14.54	4.07
*P*		0.762	0.364	0.558	0.463	0.207	0.175	0.049	0.324
ES		0.04	0.13	0.08	−0.10	0.18	0.19	0.28	0.14
Time effect									
β		−0.06	−0.06	−0.07	−2.29	2.30	−0.54	−9.36	−3.39
*P*		0.361	0.278	0.274	0.017	0.120	0.296	0.200	0.403
ES		−0.13	−0.15	−0.16	−0.35	0.22	−0.15	−0.18	−0.12
Interaction effect									
β		0.05	0.12	0.08	−1.35	2.77	0.88	26.09	3.73
*P*		0.720	0.346	0.533	0.469	0.344	0.395	0.076	0.646
ES		0.05	0.16	0.09	−0.10	0.13	0.12	0.25	0.06

The interaction (*P *< 0.1), time, and condition (both *P *< 0.05) effects are derived from mixed‐effects models. Abbreviations: β, beta coefficient; BF_mean_, mean blood flow; *D*_dia_, diastolic diameter; *D*_mean_, mean diameter; *D*_sys_, systolic diameter; ES, Cohen's *d* effect size; SR, shear rate; *V*_max_, maximum blood flow velocity; *V*_mean_, mean blood flow velocity; *V*_min_, minimum blood flow velocity.

**TABLE 2:** Seated blood pressure responses to the 6.5 h flight simulation in control‐static seating (CON) and Healthy Motion Seating (HMS).

		SBP (mmHg)	DBP (mmHg)	MAP (mmHg)	PP (mmHg)	HR (bpm)	cSBP (cm/s)	Aix (%)	AIx@75 (%)	CO (L/min)	PVR (s mmHg/mL)	aPWV (m/s)	Cdia (cm)
Mean													
CON	PRE	118	73	94	45	67	107.0	19.1	14.7	4.8	1.21	6.1	36.4
	POST	121	72	94	48	68	107.0	15.6	11.4	5.2	1.11	6.1	36.4
EXP	PRE	120	76	96	44	69	108.0	17.0	13.7	4.9	1.21	5.9	36.9
	POST	121	75	96	46	68	101.0	16.3	13.0	4.7	1.12	5.5	36.5
Standard deviation													
CON	PRE	14	9	10	12	8	14.3	11.6	11.3	0.9	0.21	1.9	3.5
	POST	14	9	10	12	4	10.3	8.4	9.0	0.7	0.18	1.6	5.4
EXP	PRE	13	9	9	13	9	11.4	12.4	12.7	0.9	0.17	1.4	3.5
	POST	13	9	10	9	8	28.4	12.3	11.1	1.4	0.36	2.1	3.3
Condition effect													
β		−0.44	−0.07	−0.36	−0.71	−0.41	−2.28	1.63	1.76	−0.21	0.02	−0.12	−0.22
*P*		0.796	0.935	0.719	0.681	0.617	0.532	0.258	0.193	0.337	0.63	0.636	0.561
ES		−0.03	−0.01	−0.04	−0.05	−0.06	−0.08	0.14	0.16	−0.12	0.06	−0.06	−0.07
Time effect													
β		1.74	−1.11	0.29	2.78	−0.01	−2.68	−1.43	−1.29	0.13	−0.08	−0.06	−0.22
*P*		0.309	0.203	0.770	0.113	0.991	0.463	0.319	0.336	0.547	0.091	0.814	0.572
ES		0.13	−0.16	0.04	0.20	0.00	−0.09	−0.12	−0.12	0.07	−0.21	−0.03	−0.07
Interaction effect													
β		−1.29	−0.61	−0.98	−0.84	−1.52	−5.41	4.10	3.92	−0.53	0.04	−0.16	−0.40
*P*		0.706	0.725	0.617	0.81	0.348	0.459	0.153	0.147	0.215	0.665	0.756	0.6
ES		−0.05	−0.04	−0.06	−0.03	−0.12	−0.09	0.18	0.18	−0.15	0.05	−0.04	−0.06

The interaction (*P *< 0.1), time, and condition (both *P *< 0.05) effects are derived from mixed‐effects models. Abbreviations: AIx, augmentation index; AIx@75, augmentation index at a heart rate of 75 beats per minute; aPWV, aortic pulse wave velocity; β, beta coefficient; CC, calf circumference; cSBP, central systolic blood pressure; CO, cardiac output; DBP, diastolic blood pressure; ES, Cohen's *d* effect size; HR, heart rate; MAP, mean arterial pressure; PP, pulse pressure; PVR, peripheral vascular resistance; SBP, systolic blood pressure; RM, reflection magnitude.

Raw data and mixed‐model findings for mood disturbance, pain and discomfort are presented in Table 3. There were significant moderate time effects for mood disturbance (7.4; 95% CI = 3.8, 10.9; *P* < 0.001, ES 0.50) and pain (7.5; 95% CI = 4.7, 10.3; *P* < 0.001, ES 0.77), and a large time effect for discomfort (8.9; 95% CI = 6.4, 11.5; *P* < 0.001, ES 0.99), in response to the 6.5 h flight simulation (all *P* < 0.05). Although there was a tendency for mood disturbance (CON vs. HMS: 191% vs. 152%), pain (CON vs. HMS: +310% vs. 244%) and discomfort (CON vs. HMS: +414% vs. 326%) to worsen to a greater degree in CON than HMS, there were no significant interaction nor group effects. Sub‐category data obtained for mood disturbance, pain and discomfort are available in the Supporting information (Tables S3–S5).

**TABLE 3:** Mood disturbance, pain and discomfort responses to the 6.5 h flight simulation in control static seating (CON) and Healthy Motion Seating (HMS).

		Mood disturbance	Pain	Discomfort
Mean				
CON	PRE	3.9	2.7	2.3
	POST	12.1	11.1	12.2
EXP	PRE	4.7	2.7	2.6
	POST	11.3	9.3	10.4
Standard deviation				
CON	PRE	15.2	3.4	3.3
	POST	16.8	11.0	9.0
EXP	PRE	26.8	2.8	3.3
	POST	27.5	12.2	11.0
Condition effect				
β		−0.77	−0.94	−1.05
*P*		0.671	0.510	0.422
ES		−0.06	−0.10	−0.12
Time effect				
β		7.40	7.50	8.92
*P*		<0.001	<0.001	<0.001
ES		0.59	0.77	0.99
Interaction effect				
β		−1.63	−1.84	−2.11
*P*		0.654	0.517	0.421
ES		−0.07	−0.09	−0.12

The interaction (*P *< 0.1), time, and condition (both *P *< 0.05) effects are derived from mixed‐effects models. Abbreviations: β, beta coefficient; ES, Cohen's *d* effect size.

## 4 DISCUSSION

The aim of this study was to determine the impact of an innovative airplane passenger seat feature, aerospace HMS, on lower‐limb blood flow and blood pressure response to simulated long‐haul air travel. The principal finding is that in response to the 6.5 h air travel simulation, lower‐limb blood flow was reduced in the traditional standard static aerospace seating but maintained in aerospace HMS. Peripheral and central blood pressure were unchanged across the flight simulation. Our findings support the notion that passive periodic alterations in sitting interface pressure, posture and movement created by aerospace HMS can protect against the deleterious effects of prolonged sitting, inherent to long‐haul air travel, on local lower‐limb blood flow.

### 4.1 Limitations and strengths

To fully contextualize the findings of this study, it is first important to acknowledge the strengths and potential limitations. Firstly, despite efforts to recruit participants across a broad age range we were only able to recruit a relatively young cohort (mean age 36 ± 15 years), which may reduce the generalizability of our findings. However, although older or at‐risk adults (e.g., those with hypertension or diabetes) may be more sensitive to prolonged uninterrupted sitting (Dempsey et al., 2018), the protective effects of its interruption appear more pronounced compared to their younger healthier counterparts (Dempsey et al., 2016; Larsen et al., 2014), suggesting that aerospace HMS could be most beneficial in these population groups. Secondly, although we successfully recruited a balanced sample (42% female), our experimental design did not account for the menstrual cycle (Wenner & Stachenfeld, 2020). However, recent findings suggest that menstrual cycle and oral contraceptive pill phases may not influence sitting‐induced changes in vascular function (O'Brien et al., 2020). Finally, our flight simulation replicated the seating and time exposures of a long‐haul flight travel only and not its hypobaric environment which can additionally challenge the cardiovascular system (Trammer et al., 2024). A significant strength of the study is its use of a randomized crossover design and control of possible effectors of lower limb blood flow and blood pressure including hydration and nutrition.

### 4.2 Comparison to the literature

Vascular homeostasis, and therefore cardiovascular health, is partly governed by the vascular endothelium (Cahill & Redmond, 2016). Bioavailability of nitric oxide is critical to endothelial function, regulating vasodilation and opposing atherogenesis. Low or turbulent flow, and subsequently shear stress, diminishes nitric oxide production, promoting endothelial dysfunction, a pro‐atherogenic environment and ultimately vascular disease (Gimbrone & Garcia‐Cardena, 2016). Low blood flow also promotes compensatory sympathetically driven vasoconstriction of blood vessels, increasing total peripheral resistance and elevating blood pressure (Dempsey et al., 2018, 2016). A novel finding of the present study is that reductions in lower‐limb blood flow induced by prolonged sitting typical of long‐haul air travel can be prevented using aerospace HMS. These findings extend the sparse HMS literature, with Dugan et al. (2009) also reporting attenuated sitting‐induced reductions in blood‐flow velocity in the lower limbs with automobile HMS, albeit with a much shorter sitting exposure period. The attenuated reductions in blood flow are likely due to the movement and subsequently alterations in posture and sitting interface pressure created as the aerospace HMS periodically extends and flexes the hips and knees, by raising and lowering the torso and lower legs, respectively (Dugan et al., 2009; Tepe & Zhang, 2011). Brief, passive movement of the lower limbs has been shown to transiently elevate blood flow in the legs (Trinity et al., 2011) as well as preserve vascular function following 2.5 h of prolonged sitting (Park et al., 2022). Mechanical compression of the vasculature stimulating shear and nitric oxide release has been proposed as potential mechanisms (Park et al., 2022; Trinity et al., 2011), although it is recognized that the frequency of movement reported in these studies is greater than that of aerospace HMS. Lower limb blood flow is also markedly affected by posture, being significantly lower in a seated position (Delis et al., 2000). This phenomenon is thought to be driven by autoregulatory vasoconstrictor mechanisms responding to vascular transmural pressure elevation and the higher hydrostatic load (Delis et al., 2000). Aerospace HMS periodically shifts a passenger between sitting and semi‐recumbent positions, with alternating loading and unloading of orthostatic stress, whereas standard static aerospace seating predominantly remains in a more acute sitting position. Finally, greater tilt and recline angles, as that cyclically created by aerospace HMS, have been shown to elicit a decrease in sitting interface pressure and a concomitant increase in ischial blood flow compared to acute sitting (Zemp et al., 2019). The aforementioned actions likely act in combination to modulate circulation, allowing for maintenance of blood flow with aerospace HMS.

Brachial and central blood pressure, as well as indices of central haemodynamics, were unchanged across the 6.5 h long‐haul air travel flight simulation (all *P* > 0.05). This finding is in slight contrast to the acute sedentary behaviour literature, with recent meta‐analyses reporting that prolonged uninterrupted sitting (modal duration of 3 h) has been reported to significantly increase SBP (Paterson et al., 2020), with a positive association between sitting time and SBP (Adams et al., 2024). However, prolonged uninterrupted sitting has not always been shown to increase SBP or mean arterial pressure in healthy adults, likely due to sufficient compensatory haemodynamic regulation in this population (Credeur et al., 2019; Kowalsky et al., 2019). A lack of change in blood pressure may also be due to the semi‐recumbent postural positions regularly adopted during the simulation, which were necessary to replicate long‐haul air travel, being less acute than that typically adopted in prolonged sitting studies (Paterson et al., 2022). Arterial angulation created by an acute sitting position, with hips and knees bent to 90 degrees, can markedly reduce popliteal artery blood flow (∼ 45%) compared with lying down in a straight body position (Morishima et al., 2017; Padilla & Fadel, 2017). That blood pressure and central haemodynamics were unaffected suggests that the physiological impact of the passive movement, postural and sitting interface pressure changes created by aerospace HMS is localized to the lower limbs. Consistent with this finding, passive lower‐limb movement has been shown to elevate femoral blood flow without changes in central blood pressure or cardiac output, emphasising that peripheral vasodilatory factors can function without significant concomitant central hemodynamic response (Venturelli et al., 2012).

Mood, pain and discomfort all significantly worsened following the 6 h long‐haul air travel flight simulation. Although there was a tendency for participants’ perceptions of mood, pain and discomfort to worsen to a greater extent in control static seats compared to aerospace HMS, no significant interactions were observed. In the only other study to examine the utility of HMS, Dugan et al. (2009) reported that following 3 h of prolonged sitting protocol, there was a tendency for mood disturbance to be lower (∼52%), whilst perceptions of total musculoskeletal pain were significantly lower (∼46.5%) in automobile HMS compared to a standard static seating (Dugan et al., 2009). The slight contrast in findings may be due to the differing seat designs. Compression at the human‐seat interface and static lordotic pelvic postures principally contribute to prolonged sitting intolerance, pain and discomfort (Brienza et al., 1996; Vergara & Page, 2002). These exposures may be greater in automobile compared to aerospace seating, with the latter offering greater reclination and lumbar/lower‐limb support, potentially augmenting the beneficial effect of HMS in automobile seating. Expectantly, passive movement of the pelvis with periodic changes in lumbar lordosis angle and human–seat interface pressure can alleviate sitting induced pain and discomfort (Reinecke et al., 1994). The attenuated pain disturbances in response to prolonged sitting in automobile HMS were attributed to the changes in seat back and seat pan‐pressure distribution it induces (Dugan et al., 2009) and a reduction in lumbar fatigue (Tepe & Zhang, 2011). But objective evaluations of seat comfort and pain over time are complex and highly subjective (Wegner et al., 2020), being influenced by seat characteristics as well as an individual's psychological and physical factors (Hiemstra‐van Mastrigt et al., 2015). This inherent variability may have masked the impact of aerospace HMS on mood, comfort and pain. Indeed, collectively, the prior literature (Dugan et al., 2009; Reinecke et al., 1994; Tepe & Zhang, 2011) suggest that the alterations in sitting interface pressure and distribution in combination with movement of the pelvis and changes in posture that aerospace HMS creates are likely to help alleviate the pain and discomfort typically experienced during long‐haul air travel.

### 4.3 Implications and perspectives

With global passenger traffic expected to more than double by 2050, with particular growth in long‐haul and ultra long‐haul travel, there is an imperative need to identify viable strategies which can attenuate the expected detrimental impact of prolonged sitting inherent to extended flight durations on passenger well‐being and experience. This study extends the literature by demonstrating that aerospace HMS, an innovative airplane seat feature which periodically alters a passenger's posture to induce movement and create alterations in sitting interface pressure, can prevent the decline in lower‐limb blood flow during long‐haul flight simulation. These findings may be particularly relevant to those with compromised cardiovascular health, given that venous stasis and reduced blood flow are principal mechanisms for venous thromboembolism (Sabanoglu, 2021; Tourn et al., 2025). However, future work will be needed to confirm the extent to which individual differences (e.g., age and disease status) impact the beneficial effects of aerospace HMS on cardiovascular health responses to long‐haul travel. A notable advantage of aerospace HMS is that existing aviation approved seating was adapted to include healthy motion technology actuation systems in the present study, adding no weight to seat infrastructure, meaning that the technology can be readily adopted in the aerospace sector.

### 4.4 Conclusions

The aim of this study was to determine the impact of an innovative airplane passenger seat feature, aerospace HMS, on lower‐limb blood flow and blood pressure response to simulated long‐haul air travel. The passive periodic alterations in sitting interface pressure, posture and movement created by aerospace HMS were protective against reductions in local lower‐limb blood flow during simulated long‐haul air travel.

## AUTHOR CONTRIBUTIONS

Keeron Stone, Barry J. Mcdonnell, Jane Lewis and Peter Sykes conceived and designed the research; Keeron Stone, Barry J. Mcdonnell, Jane Lewis, Mark Butlin, Edward Johnston, Amira Tairi, Thomas Griffiths and Bentley Gisele performed the experiments; Keeron Stone, Barry J. Mcdonnell, Jane Lewis, Mark Butlin, Edward Johnston and Thomas Griffiths analysed and interpreted the data; Keeron Stone and Jane Lewis drafted the initial manuscript; all authors reviewed and edited draft manuscripts. All authors have read and approved the final version of this manuscript and agree to be accountable for all aspects of the work in ensuring that questions related to the accuracy or integrity of any part of the work are appropriately investigated and resolved. All persons designated as authors qualify for authorship, and all those who qualify for authorship are listed.

## CONFLICT OF INTEREST

There are no conflicts of interest.

## Supporting information



Supporting information

## Data Availability

The data contained within will be made available upon reasonable request.
