# Supplementary material for: The impact of healthy motion seating on lower‐limb blood flow and blood pressure response to simulated long‐haul air travel
Source: Exp Physiol. 2025 Nov 4;111(3):1513–23. doi: 10.1113/EP092920 (PMC12949107; doi:10.1113/EP092920)
Supplement: Supplementary file 1 — Supporting information [file EPH-111-1513-s001.docx]

# TITLE: The Impact Of Healthy Motion Seating On Lower-Limb Blood Flow And Peripheral Blood Pressure Response To Simulated Long-Haul Air Travel.

**SUPPLEMENT**

**Running Title:**

Jane LEWIS^1,2^, Barry J. MCDONNELL^1,2^, Mark BUTLIN^3^, Edward JOHNSTON^1,4^, Amira TAIRI^3^, Thomas GRIFFITHS^1,4^, Gisele BENTLEY^3^, Peter SYKES^1^, Keeron STONE^1,2^.

1. Centre for Cardiovascular Research Innovation and Development, Cardiff Metropolitan University, Cardiff, Wales, United Kingdom.
2. National Cardiovascular Research Network, Wales, UK.
3. Faculty of Medicine, Health and Human Sciences, Macquarie University.
4. College of Biomedical and Life Sciences, Cardiff University, UK.

*Corresponding Author: Keeron Stone. Cardiff School of Sport and Health Sciences, Cardiff Metropolitan University, Cardiff, Wales, UK. **Tel:** 02920417139. **E:** kstone@cardiffmet.ac.uk.

# SUPPLEMENTAL TEXT

## HEALTHY MOTION SEATING POSITION ALGORITHM

**Taxi, take off, and landing position 2 (TTL2) algorithm.** The motion algorithm in the TTL2 position is composed of 6 seat back movements and 6 leg rest motions (**Table S1**). A movement occurs every 2.5 minutes. Each seat back motion moved the seat 2° with the first three motions bringing the seat back forward 6° and the final 3 motions returning it to the TTL2 home position. To better support the legs, the first leg rest motion elevated the legs 35°. Next, the legs are elevated 6° more and then returned to the 35° elevated position. This is repeated once more before returning the legs back to the TTL2 position. All leg rest motions occur during the first half of the algorithm, before the seat back begins its return to the TTL2 home position. Each change of the seat position took approximately 1.5 seconds. The TTL2 motion algorithm took 30 minutes to complete before starting again.

**Table S1.** Healthy Motion Seating movement algorithm in the taxi, take off, and landing position 2 (TTL2).

| **Time**  **(mm:ss)** | **Seat Back** | |  | **Leg Rest** | |
| --- | --- | --- | --- | --- | --- |
|  | Relative | Absolute |  | Relative | Absolute |
| 0:05 |  | +35° |  | +35° |  |
| 2:35 | +2° |  |  |  |  |
| 5:05 |  | +6° |  | +6° |  |
| 7:35 |  | -6° |  | -6° |  |
| 10:05 | +2° |  |  |  |  |
| 12:35 |  | +6° |  | +6° |  |
| 15:05 |  | -6° |  | -6° |  |
| 17:35 | +2° |  |  |  |  |
| 20:05 |  | -35° |  | -35° |  |
| 22:35 | -2° |  |  |  |  |
| 25:05 | -2° |  |  |  |  |
| 27:35 | -2° |  |  |  |  |
| 30:05 | ***(Algorithm continues with 0:05 motions)*** | | | | |

**The Lounge (Lazy Z) algorithm.** When the algorithm was started from the Lounge position, the seat back moved in 2° increments once every 3 minutes (**Table S2**). The seat back moved at first to 6° reclined, next moving to 6° inclined, before returning to the Lounge home position. Leg rest motions are closely tied to the seat back movements in this algorithm. The first seat back motion is accompanied by a +6° leg rest motion. A -6° leg rest move occurs simultaneously with the next seat back motion. There is no leg rest motion with the third seat back motion. This pattern repeats for every 3 seat back moves. Each change of the seat position took approximately 1.5 seconds. The twelve seat back motions occurred once every 3 minutes and the algorithm took 36 minutes before repeating.

**Table S2.** CMG Healthy Motion Seating™ movement algorithm in the Lounge position (Lazy Z).

| **Time**  **(mm:ss)** | **Seat Back** | |  | **Leg Rest** | |
| --- | --- | --- | --- | --- | --- |
|  | Relative | Absolute |  | Relative | Absolute |
| 0:05 | -2° | +6° |  | +6° |  |
| 3:05 | -2° | +6° |  | +6° |  |
| 6:05 | -2° | - |  | - |  |
| 9:05 | +2° | -6° |  | -6° |  |
| 12:05 | +2° | -6° |  | -6° |  |
| 15:05 | +2° | - |  | - |  |
| 18:05 | +2° | -6° |  | -6° |  |
| 21:05 | +2° | -6° |  | -6° |  |
| 24:05 | +2° | - |  | - |  |
| 27:05 | -2° | +6° |  | +6° |  |
| 30:05 | -2° | +6° |  | +6° |  |
| 33:05 | -2° | - |  | - |  |
| 36:05 | ***(Algorithm continues with 0:05 motions)*** | | | | |

## PREPARATION OF TEST SEATS FOR HEALTHY MOTION SEATING

Prior to conducting the test, it was necessary to configure two (2) of the four (4) Thompson Vantage test seats with the Healthy Motion Seating technology. The other two seats were the control seats in the study and required no modification. The following steps were performed to prepare the seats: (i) replaced the existing seat actuation system (the control unit (ECU), the three (3) seat actuation motors, and certain cables) with the Astronics PGA Gen VII actuation system. This version of the actuation system has the necessary capability to make the small, slow, smooth seat position changes required for the HMS feature that would not be possible with earlier-generation actuation systems. (ii) Comfort Motion Global’s engineer defined the motion algorithms (i.e., the seat “home” position and the sequence of and timing of seat positional changes to be executed during the test) for the TTL2 and Lounge positions. (iii) Astronics PGA’s engineer programmed the HMS algorithms into the ECU of the seat actuation system.

**Table S3.** Profile of mood state (POMS) responses to the 6.5-hour flight simulation in control static seating (CON) and Healthy Motion Seating (HMS).

|  |  | Tension-Anxiety | Depression | Anger-Hostility | Fatigue | Confusion-Bewilderment | Vigour-Activity | **Total Mood Disturbance** |
| --- | --- | --- | --- | --- | --- | --- | --- | --- |
| **Mean** |  |  |  |  |  |  |  |  |
| CON | PRE | 4.4 | 1.6 | 1.4 | 4.1 | 2.1 | 9.6 | 3.9 |
|  | POST | 5.0 | 2.2 | 1.6 | 6.7 | 2.7 | 6.0 | 12.1 |
| HMS | PRE | 4.8 | 2.7 | 1.4 | 3.5 | 2.3 | 9.9 | 4.7 |
|  | POST | 4.8 | 3.2 | 2.1 | 5.6 | 2.6 | 7.0 | 11.3 |
| **Standard Deviations** | | |  |  |  |  |  |  |
| CON | PRE | 2.3 | 2.9 | 1.8 | 4.7 | 2.0 | 7.7 | 15.2 |
|  | POST | 3.3 | 4.2 | 2.5 | 4.7 | 2.6 | 5.8 | 16.8 |
| HMS | PRE | 4.3 | 9.4 | 4.2 | 4.4 | 3.4 | 6.3 | 26.8 |
|  | POST | 4.5 | 9.0 | 3.9 | 5.6 | 3.6 | 6.8 | 27.5 |
| **Condition Effect** | | |  |  |  |  |  |  |
|  | β | -0.23 | -0.02 | 0.24 | -0.36 | -0.12 | 0.38 | -0.77 |
|  | P | 0.548 | 0.962 | 0.502 | 0.546 | 0.7 | 0.538 | 0.671 |
|  | ES | -0.09 | -0.01 | 0.10 | -0.09 | -0.06 | 0.09 | -0.06 |
| **Time Effect** | | |  |  |  |  |  |  |
|  | β | 0.26 | 0.53 | 0.45 | 2.42 | 0.45 | -3.29 | 7.40 |
|  | P | 0.484 | 0.175 | 0.217 | **<0.001** | 0.15 | **<0.001** | **<0.001** |
|  | ES | 0.10 | 0.20 | 0.18 | 0.59 | 0.21 | -0.78 | 0.59 |
| **Interaction Effect** | | |  |  |  |  |  |  |
|  | β | -0.53 | -0.11 | 0.47 | -0.53 | -0.26 | 0.68 | -1.63 |
|  | P | 0.484 | 0.891 | 0.512 | 0.661 | 0.669 | 0.577 | 0.654 |
|  | ES | -0.10 | -0.02 | 0.10 | -0.06 | -0.06 | 0.08 | -0.07 |

The interaction (P<0.1), time, and condition (both P<0.05) effects are derived from mixed-effects models. **Abbreviations:** β, beta coefficient; P, probability value; ES, Cohen’s d effect size

**Table 4.** Pain (McGill) responses to the 6.5-hour flight simulation in control-static seating (CON) and Healthy Motion Seating (HMS).

|  |  | Continuous | Intermittent | Neuropathic | Affective | **Total** |
| --- | --- | --- | --- | --- | --- | --- |
| **Mean** | |  |  |  |  |  |
| CON | PRE | 0.7 | 0.1 | 0.9 | 1.1 | 2.7 |
|  | POST | 4.7 | 0.5 | 3.5 | 2.4 | 11.1 |
| CMG | PRE | 0.9 | 0.1 | 0.8 | 1.0 | 2.7 |
|  | POST | 4.4 | 0.2 | 2.8 | 2.0 | 9.3 |
| **Standard Deviations** | | |  |  |  |  |
| CON | PRE | 1.3 | 0.2 | 1.8 | 1.9 | 3.4 |
|  | POST | 5.3 | 1.3 | 3.4 | 2.9 | 11.0 |
| CMG | PRE | 1.5 | 0.2 | 1.1 | 1.6 | 2.8 |
|  | POST | 6.7 | 0.7 | 3.2 | 2.8 | 12.2 |
| **Condition Effect** | | |  |  |  |  |
|  | β | -0.27 | -0.18 | -0.31 | -0.22 | -0.94 |
|  | P | 0.731 | 0.293 | 0.464 | 0.504 | 0.51 |
|  | ES | -0.05 | -0.15 | -0.11 | -0.10 | -0.10 |
| **Time Effect** | |  |  |  |  |  |
|  | β | 3.76 | 0.29 | 2.29 | 1.16 | 7.50 |
|  | P | **<0.001** | 0.1 | **<0.001** | **<0.001** | **<0.001** |
|  | ES | 0.70 | 0.24 | 0.78 | 0.52 | 0.77 |
| **Interaction Effect** | | |  |  |  |  |
|  | β | -0.47 | -0.37 | -0.58 | -0.42 | -1.84 |
|  | P | 0.76 | 0.293 | 0.495 | 0.517 | 0.517 |
|  | ES | -0.04 | -0.15 | -0.10 | -0.09 | -0.09 |

The interaction (P<0.1), time, and condition (both P<0.05) effects are derived from mixed-effects models. **Abbreviations:** β, beta coefficient; P, probability value; ES, Cohen’s d effect size.

**Table S5.** Discomfort responses to the 6.5-hour flight simulation in control-static seating (CON) and Healthy Motion Seating (HMS).

|  |  | **Upper Back** | **Lower Back** | **Sitting Bones** | **Buttocks Area** | **Edge of Seat Contact** | **Overall** |
| --- | --- | --- | --- | --- | --- | --- | --- |
| **Mean** | |  |  |  |  |  |  |
| CON | PRE | 0.4 | 0.4 | 0.2 | 0.2 | 0.1 | 2.3 |
|  | POST | 1.0 | 1.5 | 1.4 | 1.4 | 0.7 | 12.2 |
| HMS | PRE | 0.2 | 0.4 | 0.3 | 0.3 | 0.2 | 2.6 |
|  | POST | 0.6 | 1.2 | 1.3 | 1.3 | 0.2 | 10.4 |
| **Standard Deviations** | | |  |  |  |  |  |
| CON | PRE | 0.5 | 0.6 | 0.6 | 0.5 | 0.3 | 3.3 |
|  | POST | 1.1 | 1.1 | 1.4 | 1.4 | 1.1 | 9.0 |
| HMS | PRE | 0.5 | 0.5 | 0.6 | 0.5 | 0.7 | 3.3 |
|  | POST | 0.9 | 1.2 | 1.5 | 1.2 | 0.7 | 11.0 |
| **Condition Effect** | | |  |  |  |  |  |
|  | β | -0.10 | -0.16 | -0.11 | -0.08 | -0.29 | -1.05 |
|  | P | 0.472 | 0.326 | 0.542 | 0.624 | **0.012** | 0.422 |
|  | ES | -0.10 | -0.14 | -0.09 | -0.07 | -0.37 | -0.12 |
| **Time Effect** | |  |  |  |  |  |  |
|  | β | 0.55 | 0.90 | 1.11 | 1.13 | 0.34 | 8.92 |
|  | P | **<0.001** | **<0.001** | **<0.001** | **<0.001** | **<0.001** | **<0.001** |
|  | ES | 0.59 | 0.81 | 0.89 | 0.98 | 0.44 | 0.99 |
| **Interaction Effect** | | |  |  |  |  |  |
|  | β | -0.16 | -0.14 | -0.21 | -0.16 | -0.58 | -2.11 |
|  | P | 0.562 | 0.326 | 0.559 | 0.639 | **0.013** | 0.421 |
|  | ES | -0.08 | -0.06 | -0.09 | -0.07 | -0.37 | -0.12 |

The interaction (P<0.1), time, and condition (both P<0.05) effects are derived from mixed-effects models. **Abbreviations:** β, beta coefficient; P, probability value; ES, Cohen’s d effect size.
